# Pharmacogenomic analysis in adrenocortical carcinoma reveals genetic features associated with mitotane sensitivity and potential therapeutics

**DOI:** 10.3389/fendo.2024.1365321

**Published:** 2024-05-08

**Authors:** Jie Zhang, Luming Wu, Tingwei Su, Haoyu Liu, Lei Jiang, Yiran Jiang, Zhiyuan Wu, Lu Chen, Haorong Li, Jie Zheng, Yingkai Sun, Hangya Peng, Rulai Han, Guang Ning, Lei Ye, Weiqing Wang

**Affiliations:** ^1^ Department of Endocrine and Metabolic Diseases, Shanghai Institute of Endocrine and Metabolic Diseases, Shanghai National Clinical Research Center for Metabolic Diseases, Key Laboratory for Endocrine and Metabolic Diseases of the National Health Commission of the PR China, Shanghai Key Laboratory for Endocrine Tumor, State Key Laboratory of Medical Genomics, Ruijin Hospital, Shanghai Jiao Tong University School of Medicine, Shanghai, China; ^2^ Department of Interventional Radiology, Ruijin Hospital, Shanghai Jiao Tong University School of Medicine, Shanghai, China; ^3^ Department of Urology, Ruijin Hospital, Shanghai Jiao Tong University School of Medicine, Shanghai, China

**Keywords:** mitotane, adrenocortical carcinoma, patient-derived cells, genetic analysis, high-throughput screening

## Abstract

**Background:**

Adrenocortical carcinoma (ACC) is an aggressive endocrine malignancy with limited therapeutic options. Treating advanced ACC with mitotane, the cornerstone therapy, remains challenging, thus underscoring the significance to predict mitotane response prior to treatment and seek other effective therapeutic strategies.

**Objective:**

We aimed to determine the efficacy of mitotane via an *in vitro* assay using patient-derived ACC cells (PDCs), identify molecular biomarkers associated with mitotane response and preliminarily explore potential agents for ACC.

**Methods:**

*In vitro* mitotane sensitivity testing was performed in 17 PDCs and high-throughput screening against 40 compounds was conducted in 8 PDCs. Genetic features were evaluated in 9 samples using exomic and transcriptomic sequencing.

**Results:**

PDCs exhibited variable sensitivity to mitotane treatment. The median cell viability inhibition rate was 48.4% (IQR: 39.3-59.3%) and -1.2% (IQR: -26.4-22.1%) in responders (n=8) and non-responders (n=9), respectively. Median IC50 and AUC were remarkably lower in responders (IC50: 53.4 µM vs 74.7 µM, P<0.0001; AUC: 158.0 vs 213.5, P<0.0001). Genomic analysis revealed *CTNNB1* somatic alterations were only found in responders (3/5) while *ZNRF3* alterations only in non-responders (3/4). Transcriptomic profiling found pathways associated with lipid metabolism were upregulated in responder tumors whilst *CYP27A1* and *ABCA1* expression were positively correlated to *in vitro* mitotane sensitivity. Furthermore, pharmacologic analysis identified that compounds including disulfiram, niclosamide and bortezomib exhibited efficacy against PDCs.

**Conclusion:**

ACC PDCs could be useful for testing drug response, drug repurposing and guiding personalized therapies. Our results suggested response to mitotane might be associated with the dependency on lipid metabolism. *CYP27A1* and *ABCA1* expression could be predictive markers for mitotane response, and disulfiram, niclosamide and bortezomib could be potential therapeutics, both warranting further investigation.

## Introduction

Adrenocortical carcinoma (ACC) is a rare but fatally aggressive endocrine malignancy with high risk of recurrence and dismal prognosis (1, 2). However, therapeutic options for advanced ACC are limited. Mitotane, a derivative of insecticide dichlorodiphenyltrichloroethane (DDT) with adrenolytic properties, has been currently the only drug approved by the U.S. Food and Drug Administration (FDA) and European Medicines Agency (EMA) for ACC (1, 3, 4). Mitotane alone or in combination with platinum-based chemotherapy is recommended as first-line therapy in the palliative setting for advanced and unresectable tumors as well as in adjuvant settings in patients at high risk of recurrence (5, 6).

Despite over five-decade application in clinics, treatment with mitotane remains challenging. Firstly, the dose-limiting toxicity and narrow therapeutic window of mitotane makes it a difficult drug to manage and requires personalized dosing regimen, partially due to its exceedingly poor aqueous solubility and low bioavailability (7, 8). Secondly, the action of mitotane is not immediate but latent, with time needed to attain target plasma concentrations during which disease progression may precede (9). Moreover, the response spectrum to mitotane differs between patients and the response rates were between 10% and 35% (5, 10, 11). Additionally, since mitotane is a strong inducer of *CYP3A4* with long-lasting effect, drug interactions with mitotane pose another issue (12, 13). Lastly, adverse effects including gastrointestinal, central nervous system, endocrine and hepatic toxicity would limit its tolerability and even lead to the discontinuation of treatment (6). Therefore, identifying markers to predict response to mitotane is of remarkable importance to spare unfavorable drug toxicity, time window for other treatments, and costs as well.

Efforts on determining predictive markers for mitotane response have long been made. To date, mitotane plasma levels within the target range of 14 to 20 mg/L is considered the strongest predictor of mitotane effectiveness. Plasma mitotane level above 14 mg/L was significantly associated with improved tumor response and survival (14–16). As for molecular predictors, germline CYP2W1*6 single nucleotide polymorphism was associated with a reduced probability to reach target concentration and lower response rates, whereas CYP2B6*6 correlated with higher mitotane levels (17). Other potential predictive factors include those implying mitotane action and its potential target (e.g., SOAT1) (18, 19). Theoretically, treatment response is also dictated by the intrinsic molecular state of tumors before drug exposure (20, 21). The first study on assessing the direct effects of mitotane in a large series of primary human ACC cultures has found the efficacy of mitotane was highly variable and *RRM1*, *SOAT1* as well as *CYP2W1* expression levels were not predictive for mitotane sensitivity *in vitro* (10). Hence, identifying molecular features to indicate mitotane response is urgent.

In combination with mitotane, cytotoxic chemotherapy including etoposide, doxorubicin, cisplatin (EDP-M) is recommended in first-line settings (5). EDP-M regimen prolonged progression-free survival to five months but failed to improve the overall survival (22). Nevertheless, adverse events from chemotherapy are common and diverse (6). Thus, seeking novel therapeutic strategies is urgently needed.

In this study, we conducted *in vitro* mitotane sensitivity testing to evaluate direct antitumor activity in patient-derived ACC cells (PDC) obtained from 17 patients in an attempt to distinguish the therapeutic response of mitotane through a rapid *in vitro* assay. Further, we performed genomic and transcriptomic study in order to dissect molecular profiling of mitotane responders and non-responders, aiming to identify molecular biomarkers associated with individual response to mitotane. Additionally, high-throughput screening (HTS) against 40 compounds was conducted in an effort to explore other potential agents.

## Materials and methods

### Patients and sample collection

**Figure 1:**
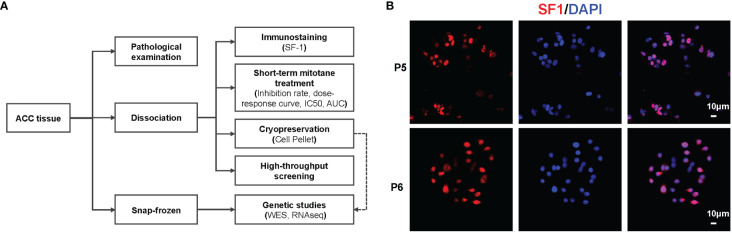
Overview of *in vitro* mitotane sensitivity testing for ACCs. **(A)** Pipeline of patient tissue processing. **(B)** Representative images of immunofluorescence staining of PDCs from P5 (Responder) and P6 (Non-responder).

Fresh primary ACC tissues were obtained from patients upon resection or biopsy at Ruijin Hospital between September 2020 to July 2023. The ACC diagnosis was confirmed by experienced pathologists, and steroidogenic factor 1 (SF1) immunostaining was performed to confirm its adrenal cortex origin. Clinicopathological information including age, sex, ENSAT stage, Ki67 index and hormonal secretion status, systemic therapies received prior to surgery or biopsy was recorded and analyzed. Hormonal secretion status was evaluated using biochemical testing of serum steroid hormone levels (e.g., cortisol, aldosterone and androgens) and 1mg dexamethasone suppression test. Informed consent was obtained from all patients, and this study was approved by local ethics committee of Ruijin Hospital (Approval number: KY320). Upon surgical or biopsy removal, pieces of tumors were fixed in formalin and paraffin-embedded for pathological diagnosis. For primary cell cultures, tumor tissues were placed in Tissue Storage Solution (Miltenyi Biotec, Cat No.130-100-008). Additional tissues were immediately snap-frozen in liquid nitrogen for later use. The overview of the tissue process pipeline was summarized in **Figure 1A**.

### Dissociation and short-term culture of PDCs

Immediately after surgery or biopsy, tumor tissues were collected in the Tissue Storage Solution (Miltenyi Biotec, Cat No.130-100-008) and transported to the laboratory on ice and isolated within 24 hours. Tumor tissues were rinsed with Hanks’ Balanced Salt Solution (HBSS; Gibco, Cat No.14175095), minced and digested with 2.0 mg/mL collagenase II (Gibco, Cat No. 17101015), 0.02 mg/mL DNase (Roche, Cat No. 11284932001) at 37°C on a shaker for up to 2 hours. Then this suspension was filtered through a 70-µm cell strainer (Falcon, Cat No.352350). After depletion of red blood cells using Red Blood Cell Lysis Buffer (Invitrogen, Cat No.00-4333-57), trypan blue staining (Gibco, Cat No.15250061) was performed for cell counting and viability assessment. After cell preparation, one portion was plated directly into 96-well plates (Corning, Cat No.3799) for mitotane sensitivity testing, whilst a small number of cells was plated in chamber slides (Millipore, Cat No. PEZGS0816) or CellCarrier Ultra plates (PerkinElmer, Cat No.6055300) for immunofluorescence staining of adrenal cortex marker, SF1 (Proteintech, Cat No.18658-1-AP). When cell amount was abundant, cells would be plated in 384-well plate for HTS and cryopreserved in liquid nitrogen for later use. Cells then were cultured in DMEM/F-12 (Gibco, Cat No. 11320033) medium supplemented with 10% fetal bovine serum (FBS; Gibco, Cat No. 10099141), 1% Penicillin-Streptomycin (10,000 U/mL; Gibco, Cat No. 15140-122) and 1% L-glutamine (200 mM; Gibco, Cat No. 25030-081).

### *In vitro* mitotane sensitivity testing

Mitotane (MedChemExpress, Cat No. HY-13690) was dissolved in dimethyl sulfoxide (DMSO, Sigma, Cat No. D2650) to a concentration of 100mM as stock solution, aliquoted and stored at -80°C. For *in vitro* experiments, the final concentration of DMSO was ≤0.1%. Primary cells were plated in a 96-well plate at a density of 1.0x10^4 cells/well in triplicates and treated with mitotane (1.0μM-100μM) for 72 hours and cell viability was assessed by the Cell Counting Kit-8 (CCK-8) assay kit (Dojindo, Cat No. CK04). Mitotane-sensitive ACC cell line H295R (ATCC^®^ CRL­2128™) was used as the positive control. Dose-response curves, inhibition rate, half maximal inhibitory concentration (IC50) values and area under dose response curve (AUC) were calculated in Prism8.3 software (GraphPad). PDCs were arbitrarily classified as non-responders when the inhibitory effect on cell viability was less than 33% at the concentration of mitotane corresponding to the therapeutic circulating plasma concentration (14 mg/L, 50 µM) according to the previous study (10).

### Immunofluorescence staining

Briefly, cells were fixed with 4% paraformaldehyde for 15 min at room temperature, and then washed twice with PBS buffer (Sangon Biotech, Cat No. B548117), followed by permeabilization with 0.1% Triton X-100 (Sigma-Aldrich, Cat No. 9036-19-5) for 15min. Next, cells were washed twice with PBS and blocked using antibody diluent (DAKO, Cat No. s3022) for 1h at room temperature. Later, cells were incubated with primary antibody against SF1 (1:100, Proteintech, Cat No.18658-1-AP) at 4°C overnight, followed by YSFluor 594-conjugated secondary antibodies (1:500, Yeasen Biotechnology, Cat No. 34212ES60). Nuclei were stained with 4,6-diamidino-2 phenylindole (DAPI) and wells were mounted using DAPI Fluoromount-G (SouthernBiotech, Cat No. 0100-20).

### High-throughput screening

Cells were plated in 384-well plates (PerkinElmer, Cat No.6007680) at a density of 2000 cells per well in 50μl total volume. HTS was performed in an automated Cell::explorer HTS pro Platform (PerkinElmer). 24 hours after seeding, cells were treated with test compounds using a robot plate::handler equipped with a pintool dispensing device (PerkinElmer) for 6 days. HTS was conducted in single with four concentrations for each compound. DMSO was used as the vehicle control. Cell viability was determined using CellTiter-Glo reagent (Promega, Cat No. G7572) and luminescence was measured on an EnVision multimode plate reader (PerkinElmer). Dose-response data were analyzed. IC50 and AUC were calculated in Prism8.3 software (GraphPad).

### DNA and RNA extraction

Genomic DNA and total RNA were extracted from snap-frozen tumor tissues or patient-derived primary cell pellets using the AllPrep DNA/RNA Micro Kit (Qiagen, Cat No. 80284) according to the manufacturer’s instructions. DNA extraction from peripheral blood leukocytes was carried out using the QIAamp DNA Mini Kit (Qiagen, Cat No. 51304). DNA and RNA concentrations were evaluated on Qubit Fluorometer (Thermo Fisher Scientific).

### Whole exome sequencing

WES was performed on the tumor DNA and matched blood DNA. Briefly, genomic DNA of tumor and paired peripheral blood samples from 9 patients was randomly sheared through ultra-sonication to generate paired-end libraries with an average insert size of ~300 bp. Exome regions were captured by the xGen Exome Hyb Panel v2 kit (Integrated DNA Technologies, Cat No. 10005153), and sequencing was performed on Illumina Novaseq 6000 platform (Illumina, San Diego, CA, USA) with 150 bp paired end strategy.

### Identification of somatic mutations

The paired-end reads from WES were mapped to human reference genome (hg19) by BWA aligner (v0.7.17) (23). Mapping results were then sorted and marked for duplications via Picard (v2.23.0) (24). Single nucleotide variants (SNVs) and small insertions and deletions (INDELs) were obtained by taking the union of three callers GATK4 Mutect2 (25), VarDict (26), and MuTect (27). All mutations were annotated by snpEff (v4.2), and ANNOVAR (v2019Dec03). All functional mutations, including missense, nonsense, splicing, nonstop SNVs, and INDELs, were obtained. Homemade pipelines were used to filter SNVs and INDELs: 1) mutations were called by more than one software; 2) variant allele frequencies (VAFs) were ≥ 10% and ≥ 4 individual mutant reads.

### Analysis of copy number variant

Sequencing coverage and copy number in the aligned sequencing reads from WES were analyzed using CNVkit (v0.9.7) (28). The sequencing coverage of WES in germline samples was assessed and used to create pooled reference data that included the technical variability at each exon region. The read depths of tumor samples were individually compared with the reference after normalization (corrected for GC content, target footprint size and spacing, and repetitive sequences). The copy number segments were inferred by the circular binary segmentation algorithm (29).

### RNA sequencing and analysis

We utilized the KAPA RNA Hyper Prep Kit (Kapa Biosystems, Cat No. KK8544) for library preparation. Sequencing was performed in the Illumina Nova S4 platform. The Illumina bcl2fastq Conversion Software was used to convert base call (BCL) files into FASTQ files. The sequences were aligned to hg38 reference genome using HISAT2 (30) and gene expression levels were quantified using RSEM (31). Count correction was performed using the removeBatchEffect function from the limma R package (32) and then the batch-corrected expression matrix that used to heatmap analysis were constructed based on log-normalized transcripts per million (TPM) of each gene. P value < 0.05 and |logFoldChange| ≥ 1.5 was set as the threshold for significantly differential expression. Gene Set Enrichment Analysis (GSEA) (33) was performed based on Gene Ontology (GO) database (34, 35) and KEGG.

### Statistical analysis

Data were presented as mean ± SEM, or as medians and interquartile ranges (IQR), whilst categorical variables were presented as percentages and absolute numbers. Statistical analysis was performed using IBM SPSS Statistics Version 26.0 and GraphPad Prism 8. All P values were two-sided, with P < 0.05 considered statistically significant.

## Results

### Establishment of ACC primary cultures

PDCs were successfully obtained from 17 ACCs upon surgery or biopsy, including six primary tumors, two local recurrent and nine metastatic tumors (lung, liver, etc.). SF1 immunofluorescence staining was performed to confirm the adrenal cortex origin of tumor cells (**Figure 1B**). Patient characteristics are listed in **Supplementary Table 1**. Of note, six patients received mitotane prior to surgical or biopsy intervention for a period of 2 months to 18 months, but underwent disease progression or lacked satisfactory response. The principal aim was to evaluate response of PDCs to mitotane and identify biomarkers to predict sensitivity (**Figure 1A**). Since the yield of the dissociation procedure varied because of differences in the size of the tumor tissue available, only when cell amount permitted, HTS would be performed to seek for other potential agents.

### ACC PDCs depict differential sensitivity to mitotane *in vitro*

**Figure 2:**
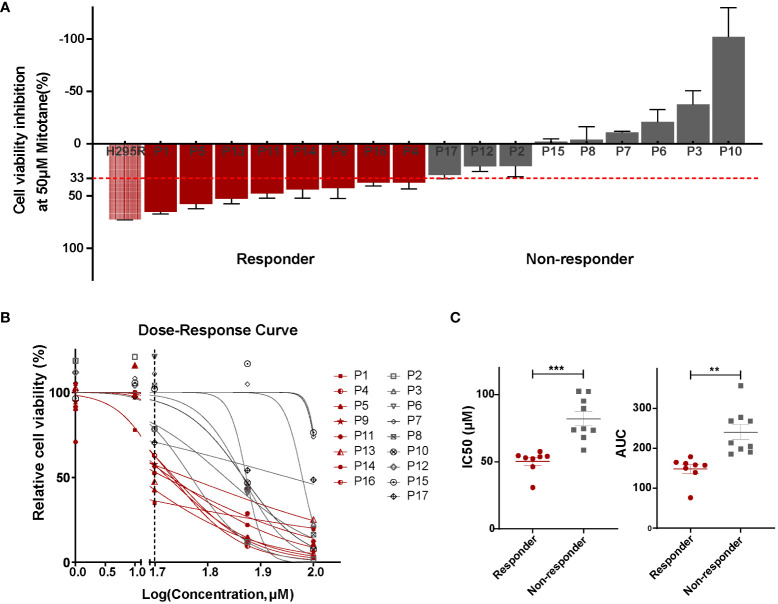
Patient-derived ACC cells depicted differential sensitivity to mitotane. **(A)** Cell viability inhibition at 50 µM mitotane for each individual primary cultures and determination of responders (red) and non-responders (grey). Mitotane-sensitive ACC cell line H295R was used as the positive control. Cells were plated in triplicates and error bars represent SEM of triplicates. **(B)** Dose–response curves of mitotane on cell viability in responders (red) and non-responders (grey). The vertical dashed line represented the concentration used for *in vitro* response classification (50 µM). **(C)** Comparison of IC50 values (left) and area under dose response curve (AUC)(right) between responders (red) and non-responders (grey). ***P < 0.001, **P < 0.01.

First of all, we performed *in vitro* sensitivity testing in PDCs for a 3-day mitotane exposure, allowing to exclude the impact of patient tolerance or pharmacokinetics. Cell viability inhibition at 50 µM mitotane is used to group ACCs into responders (>33% inhibition) and non-responders (≤33% inhibition). The median cell viability inhibition rate at 50 µM mitotane was 30.4% (IQR: -7.1%-47.9%). Eight patients (47%) were classified as responders with inhibition rate reached 48.4% (IQR: 39.3%-59.3%) whereas nine (53%) non-responders were scarcely inhibited by 50µM mitotane, with median inhibition rate of -1.2% (IQR: -26.4%-22.1%) (**Table 1**; **Figure 2A**). Dose-response curves showed the different potency of mitotane in the two groups as non-responders had higher IC50 values. Median IC50 for responders and non-responders were 53.4 µM (47.8-54.4µM) and 74.7 µM (70.9-98.8µM), respectively (P<0.0001). AUC were greatly increased compared to responders, and estimated AUCs were158.0 (142.1-164.3) and 213.5 (194.5-273.1) in responders and non-responders, respectively (P<0.0001) (**Table 1**; **Figures 2B, C**). Clinical response data were obtained from eight patients: all three non-responders showed clinical progressive disease; three responders showed clinical stable disease, while two responders progressed (**Table 2**). The consistence rate between *in vitro* test and clinical response is 75% (6/8).

**Table 1:** Comparison of patient characteristics between responders and non-responders identified by *in vitro* mitotane sensitivity testing.

	Total	Responder	Non-responder	P value
**Patient number (n, %)**	17	8 (47%)	9 (53%)	
**Age (yrs), mean ± SD**	51.5 ± 20.0	57.8 ± 12.4	45.9 ± 24.3	0.233
**Sex (Female/Male)**	12/5	7/1	5/4	0.149
ENSAT staging (n,%)				
II	3 (17.6%)	1 (12.5%)	2 (22.2%)	0.704
III	3 (17.6%)	2 (25.0%)	1 (11.1%)	
IV	11 (64.7%)	5 (62.5%)	6 (66.7%)	
Functionality (n,%)				
Functional	9 (52.9%)	6 (75.0%)	3 (33.3%)	0.086
Non-functional	8 (47.1%)	2 (25.0%)	6 (66.7%)	
Ki67 index				
NA	3	1	2	
10%-19%	6 (42.9%)	3 (42.9%)	3 (42.9%)	0.766
20%-39%	3 (21.4%)	1 (14.3%)	2 (28.6%)	
≥ 40%	5 (35.7%)	3 (42.9%)	2 (28.6%)	
*In vitro* mitotane sensitivity testing				
Inhibition at 50 µM mitotane (%), median (IQR)	30.4 (-7.1-47.9)	48.4 (39.3-59.3)	-1.2 (-26.4-22.1)	<0.0001
IC50 (µM), median (IQR)	58.7 (53.4-82.2)	53.4 (47.8-54.4)	74.7 (70.9-98.8)	<0.0001
AUC, median (IQR)	185.7 (158.0-240.8)	158.0 (142.1-164.3)	213.5 (194.5-273.1)	<0.0001

yrs, years; SD, standard deviation; IQR, interquartile range; IC50, half maximal inhibitory concentration; AUC, area under curve; NA, not available.

**Table 2:** Clinical responses of the 8 patients receiving mitotane treatment after *in vitro* test.

Patient ID	*In vitro* mitotane efficacy	Mitotane dosage	Target lesion		Clinical response
			Baseline	Post-treatment (3 month)	
P1	Responder	2g	3mm	3mm	Stable disease
P4	Responder	2g	10mm	14mm	Progressive disease
P5	Responder	3g	21mm	17mm	Stable disease
P13	Responder	3g	–	New lesion (31mm)	Progressive disease
P14	Responder	3g	3mm	3mm	Stable disease
P7	Non-responder	2g	9mm	12mm	Progressive disease
P12	Non-responder	2g	10mm	10mm with new lesions	Progressive disease
P15	Non-responder	3g	7mm	13mm	Progressive disease

### Comparison of patient characteristics between mitotane responders and non-responders

Comparison of clinicopathological characteristics between responders and non-responders demonstrated no significant differences regarding as age (57.8 ± 12.4 vs 45.9 ± 24.3, P=0.233), gender (P=0.149), ENSAT staging (P=0.704) and Ki67 index (P=0.766). Noteworthily, functional tumors with steroid hormone secretion showed a tendency of better response *in vitro* than non-functional ones (66.7% vs 25.0%, P=0.086) (**Table 1**). A negative correlation was found between tumor functionality and AUC (Spearman correlation coefficient= -0.481, P=0.051) with marginal significance, in line with above findings, indicating a tendency that tumors with active hormonal function might respond better to mitotane treatment.

### Genetic analysis discovers features associated with mitotane sensitivity *in vitro*

To identify molecular factors contributing to mitotane response, we then conducted genomic (WES) and transcriptomic sequencing (RNAseq) on tissue samples or primary cell pellets when there were no additional tissues available. In order to reveal intrinsic genetic features underlying mitotane sensitivity rather than acquired molecular features induced by mitotane treatment, a total of nine samples free from mitotane exposure were sequenced, from five responders and four non-responders classified by *in vitro* sensitivity testing.

**Figure 3:**
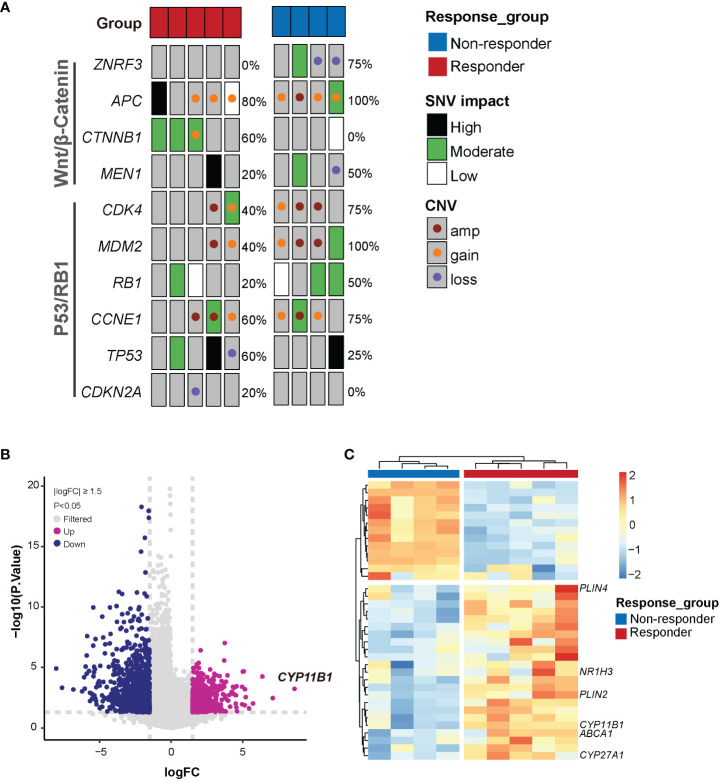
Genetic features associated with mitotane sensitivity *in vitro*. **(A)** Somatic alterations of Wnt/β-catenin and P53/RB1 pathway genes in mitotane responders and non-responders. Alteration frequencies are shown on the right side. SNV, Single nucleotide variant; CNV, copy number variant; amp, amplification. **(B)** Volcano plot of highly significant differential genes associated with response to mitotane. FC, foldchange. **(C)** Heatmap of the differentially expressed genes between responder and non-responder groups.

Alterations in the established driver genes including SNVs and CNVs were observed in P53/RB cell-cycle pathway (8/9, 88.9%) and Wnt/β-Catenin signaling pathway (9/9, 100%) (**Figure 3A**). 4/5 responders and 3/4 non-responders harbored genetic alterations in both pathways concurrently. More specifically, somatic mutations in *TP53* were found in 3 patients (2/5 responders and 1/4 non-responders) and loss of *TP53* was found in 1 responder (1/5). *RB1* mutation was identified in 1/5 responders and 2/4 non-responders. CNV gain or amplification in *CDK4*, *CCNE1* and *MDM2* were identified in 7 patients. It was well acknowledged that *CTNNB1* mutations and *ZNRF3* alterations were mutually exclusive (36). Surprisingly, we found their exclusive presence in responders and non-responders. Responder group merely harboring *CTNNB1* somatic mutations (3/5) while non-responder group presenting only *ZNRF3* alterations (3/4). Moreover, *APC* alterations were observed in 4 responders and 4 non-responders. However, whether *CTNNB1* and *ZNRF3* alterations render differential intrinsic sensitivity to mitotane requires further investigations.

**Figure 4:**
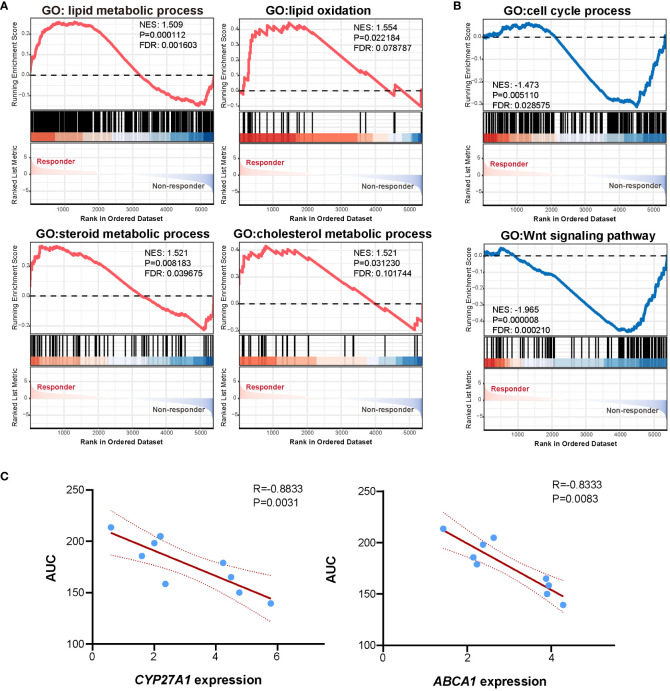
Functional enrichment and correlation analysis. **(A)** GSEA analysis showed pathways enriched in the responder group. **(B)** GSEA analysis showed pathways enriched in the non-responder group. **(C)** Scatter plot showing linear correlation between mitotane AUC and *CYP27A1* expression and *ABCA1* expression.

RNAseq was performed to investigate gene expression signatures. A total of 1612 genes were differentially expressed (|logFoldChange| ≥ 1.5, P<0.05) between responder and non-responder tumors (**Figure 3B**). Evidence has accumulated that mitotane dysregulated lipid metabolism and raised the potential correlation between mitotane responsiveness and capacity of handling lipids (37, 38). From our transcriptome data, to be noted, expression of genes involved in steroidogenesis (*CYP11B1*) and lipid metabolism (*CYP27A1, ABCA1, PLIN2, PLIN4, NR1H3*, etc) were significantly upregulated in mitotane-sensitive tumors (**Figure 3C**), implying elevated capacity for handling intracellular lipids. Consistently, functional enrichment analysis using GSEA showed pathways associated with lipid metabolism are significantly upregulated in responders and non-responders including lipid metabolic process, lipid catabolic process, lipid oxidation, cholesterol metabolic process and steroid metabolic process, etc, possibly underlying tumor functionality (**Figure 4A**). On the other hand, Wnt signaling pathway and cell cycle process were significantly downregulated in the responder group (**Figure 4B**).

To further investigate marker genes correlated to *in vitro* mitotane responsiveness, Spearman correlation analysis was performed between gene expression levels and response data of AUC. A list of genes previously reported as key regulators of lipid metabolism (uptake, biosynthesis, storage, and lipolysis, efflux, etc), steroidogenesis as well as genes priorly proposed to be potentially predictive for mitotane response were analyzed. We failed to find correlation between *RRM1*, *SOAT1*, *CYP2W1* mRNA expression level and mitotane responsiveness (**Supplementary Figure 1**). Of note, oxysterol synthetic enzyme, *CYP27A1* and cholesterol efflux pump, *ABCA1* were negatively correlated to AUC (**Figure 4C**), denoting the higher expression of *CYP27A1* and *ABCA1*, the lower of the AUC value and the better of *in vitro* responsiveness to mitotane. As a pivotal mechanism for preventing intracellular free cholesterol accumulation, it was tempting to speculate that higher *CYP27A1* and *ABCA1* implied higher intracellular free cholesterol at baseline, which required enhanced conversion and efflux ability, thus more susceptible to mitotane.

These findings indicated that the dependence on the higher capacity for lipid metabolism to maintain intracellular lipid balance conferred ACC more vulnerable to mitotane.

### Pharmacologic analysis reveals potential active agents against primary ACC cells

**Figure 5:**
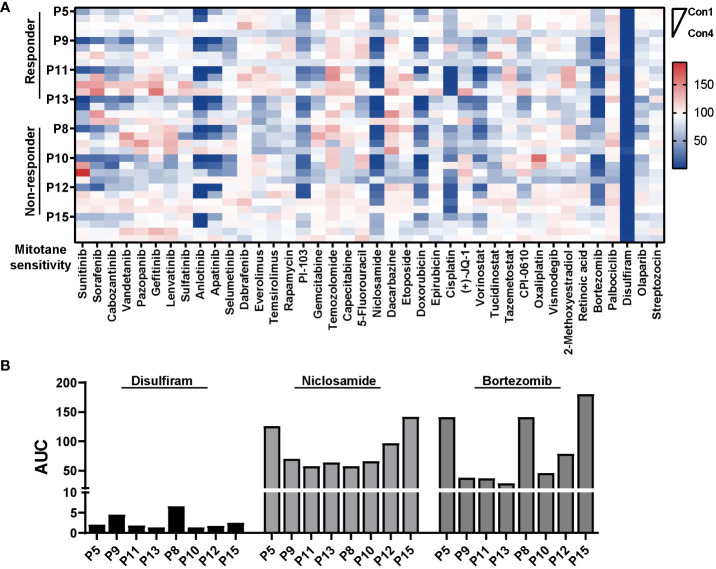
Pharmacologic analysis reveals potential active agents against PDCs. **(A)** Heatmap representation of cell viability for eight PDCs tested in a proliferative assay against 40 compounds. HTS was conducted in single with four concentrations for each compound. **(B)** Bar plot showing AUC of disulfiram, niclosamide and bortezomib in eight PDCs.

In order to uncover potential therapy for ACC especially those mitotane non-responders, we designed and set up a compound library containing 40 compounds in four concentrations. Primarily, drugs or compounds were chosen based on the following criteria: 1) The drug was FDA-approved or in clinical trials; 2) The compound had been reported effective in ACC models or proposed as potential targeted anti-cancer treatments for ACC (39–41). Drugs and highest concentrations used in HTS as well as references are listed in **Supplementary Table 2**. Aiming to establish a differential cytotoxicity assay, a 6-day treatment with compounds were performed in PDCs. Eight patient-derived ACC cells (four responders and four non-responder) were tested in a proliferative assay against our in-house library in 384-well plates. Surprisingly, both mitotane responsive and non-responsive ACC cells were extremely vulnerable to disulfiram treatment. Antihelminthic agent, niclosamide, and proteasome inhibitor, bortezomib, which were previously reported effective in ACC cell lines (42, 43), were identified efficacious in 6/8 and 5/8 PDCs, with estimated IC50 ranging from 0.22μM to 0.77μM and 10nM to 50nM, respectively. Furthermore, doxorubicin and cisplatin were effective in 3/8 and 2/8 ACCs, respectively. Additionally, PI-103, a PI3K/mTOR inhibitor, was active in 4/8 ACCs and primary culture derived from Patient 13 demonstrated sensitivity to multi-targeted tyrosine kinase inhibitor, sunitinib and anlotinib (**Figures 5A, B**).

## Discussion

It is important to identify predictive factors associated with mitotane efficacy in ACC for patient selection and seek other potential treatment. In current study, we revealed 1) variable sensitivity to mitotane in primary ACC cultures; 2) response to mitotane might be associated with the capacity for lipid metabolism; 3) potential drug repurposing opportunities for existing drugs including disulfiram, niclosamide and bortezomib.

The overall clinical efficacy of mitotane in ACC patients were 10%-35%. In our *in vitro* assay, 8 patients (47%) were classified as responders. A higher response rate *in vitro* was also observed in Dr.Hofland’s study (10). This phenomenon may be due to the fact that in clinical settings, the concentration of mitotane reaches to the therapeutic effect level in only about 50% of patients (44), while the mitotane concentration in *in vitro* test would be homologous. In a pilot cytotoxicity study, we found there was no difference between 3-day and 6-day treatment of mitotane in term of IC50 measurement, which is consistent with previous study that mitotane exerts its cellular effect within the first 24 hours *in vitro* occurred early after exposure (19). Therefore, we adopted a 3-day assay of mitotane in PDCs. The short-term culture could also avoid fibroblast outgrowth and managed to differentiate the heterogenous response.

Here a cut-off of 33% reduction in cell viability was used as an index of *in vitro* sensitivity with 50µM mitotane treatment. The consistence rate between mitotane *in vitro* sensitivity test and clinical response is 75%. A larger sample size with more *in vitro* sensitivity testing and corresponding clinical response to mitotane in the respective patients certainly would be necessary for determination of the most appropriate cut-off value. Our results indicated that a 3-day *in vitro* mitotane sensitivity testing was technically feasible for rapid mitotane response prediction.

Comparison of clinical features among responders and non-responders, we found hormonally active tumors tended to respond better to mitotane exposure *in vitro*. Six of nine functional ACCs were in the responder group. Specifically, in the responder group, functional tumors accounted for 75% (6/8) composing of four cortisol-secreting ACC with one androgen co-secretion and two androgen-secreting ACC, while in the non-responder group cortisol-secreting (2/9) and aldosterone-secreting (1/9) ACC accounted for 22.2% and 11.1%, respectively. This was consistent with Dr.Hofland’s findings that the proportion of cortisol-producing ACC was highest in the responder group (73%), with a gradually decreasing percentage from the partial responder (43%) to the non-responder group (14%, P = 0.068) (10).

Possible association between tumor hormonal activity and *in vitro* mitotane sensitivity was also implicated in transcriptomic features. Our transcriptome data revealed that *CYP11B1* was the most upregulated gene and the steroid hormone metabolic process was significantly enriched in the responder group, in support of previous discovery that the metabolic activation of mitotane is mainly dependent on *CYP11B1* (45). Elevated mRNA expression of *CYP27A1* and *ABCA1* was identified to be correlated with higher mitotane sensitivity. Mitochondrial hydroxylase CYP27A1 is a key enzyme responsible for converting cholesterol to oxysterol, namely the 27-hydroxycholesterol (27HC). It acts as liver X receptor (LXR) agonist and upregulates expression of cholesterol efflux pumps (i.e., ABCA1 and ABCG1) to prevent intracellular cholesterol accumulation (46). CYP27A1 is abundant in adrenal cortex, more pronounced in zona fasciculata (47). Oxysterol/LXR involves in adrenal steroidogenesis and serves as a safety valve to limit free cholesterol levels, thereby protecting adrenal cortex from lipotoxicity (48). Because mitotane could cause lipotoxicity in ACC cells through targeting lipolysis and cholesterol storage (19, 38), we hypothesize that ACCs expressing higher level of *CYP27A1* and *ABCA1* might tightly depend on its capacity of handling cholesterol flux, thus vulnerable to disturbance of lipid homeostasis induced by mitotane.

*CTNNB1* mutation and *ZNRF3* alterations are among the most common somatic changes in ACC (36, 49). The genomic analysis uncovered *ZNRF3* alteration in three (3/4) non-responders (3/4) and *CTNNB1* alteration in three (3/5) responders. However, to elucidate the relationship of *CTNNB1* and *ZNRF3* alteration to mitotane response needs further investigations. A higher percentage of patients harboring alterations affecting both TP53/RB and Wnt/β-Catenin pathway was observed, which might be due to the fact that patients included in this study were more aggressive with dismal outcomes (50). Additionally, a significant enrichment of Wnt signaling and cell cycle process in non-responder group was observed from transcriptomic data, indicating a more pronounced dysregulation of these two pathways. Given the relatively small sample size, these observations need more cautious interpretation.

Improved therapeutics for advanced ACC have long been the unmet medical need. Here, we used PDCs for HTS aiming to identify potential agents for ACC, particularly to explore drug repurposing chances. Patient 12 (P12) had previously received chemotherapy regimen (etoposide and carboplatin) for four cycles but suffered progressive disease. Primary cells derived from this patient showed great resistance to etoposide and oxaliplatin but sensitive to cisplatin. There was a good consistency in clinical and *in vitro* response to etoposide. However, the differential sensitivity to cisplatin and oxaliplatin, carboplatin might be attributed to different potency and mode of action of these platinum analogues (51–53). Notably, niclosamide and bortezomib were highly efficacious in PDCs with IC50 below the known maximum plasma concentration (Cmax) in human,18.34 μmol/L and 120.3 ng/ml for niclosamide and bortezomib, respectively (43, 54). Applying PDCs in drug repurposing might be a promising strategy to guide personalized therapy in ACC.

Our study has the strength of integrating genomic, transcriptomic, and pharmacological analysis of ACC PDCs to identify molecular biomarkers associated with mitotane response and performing HTS against PDCs to uncover potential active agents for the first time. Efforts have been made to identify correlations between *in vitro* mitotane response and clinical response and the consistence rate reached 75% (6/8). But still, our research has several limitations. First, there was a lack of available mitotane plasma concentrations which might be responsible for clinical progressive disease in “responders”. In six metastatic patients, clinical response to the lesions where primary culture derived could not be evaluated for they underwent locoregional treatments including surgery (one patient), radiofrequency ablation (RFA, four patients) and transarterial embolization (TAE, one patient). Second, the number of primary cultures tested were still limited because of the rarity of the ACC. A larger cohort would be required for establishment of more robust gene-drug associations.

In summary, ACC PDC models provided a feasible approach for pharmacologic sensitivity evaluation to guide personalized therapies. Clinical features and transcriptomic signatures suggested the hormonal secretion activity of ACC might be associated with response to mitotane, warranting further investigation. Future research needs to confirm whether the *CYP27A1* and *ABCA1* expression level could be used as mitotane sensitivity predictor.

## Data availability statement

The original contributions presented in the study are publicly available. This data can be found here: https://ngdc.cncb.ac.cn/gsa-human/, accession number: HRA006596.

## Ethics statement

The studies involving humans were approved by local ethics committee of Ruijin Hospital. The studies were conducted in accordance with the local legislation and institutional requirements. The participants provided their written informed consent to participate in this study.

## Author contributions

JZha: Formal analysis, Methodology, Visualization, Writing – original draft, Writing – review & editing. LW: Investigation, Resources, Writing – original draft, Writing – review & editing. TS: Investigation, Resources, Writing – review & editing. HLiu: Methodology, Visualization, Writing – review & editing. LJ: Investigation, Resources, Writing – review & editing. YJ: Investigation, Resources, Writing – review & editing. ZW: Investigation, Resources, Writing – review & editing. LC: Investigation, Resources, Writing – review & editing. HLi: Methodology, Writing – review & editing. JZhe: Methodology, Visualization, Writing – review & editing. YS: Methodology, Visualization, Writing – review & editing. HP: Methodology, Writing – review & editing. RH: Methodology, Writing – review & editing. GN: Supervision, Writing – review & editing. LY: Conceptualization, Supervision, Writing – review & editing. WW: Conceptualization, Supervision, Writing – review & editing.

## References

[1] Else T Kim AC Sabolch A Raymond VM Kandathil A Caoili EM. Adrenocortical carcinoma. Endocr Rev. (2014) 35:282–326. doi: 10.1210/er.2013-1029 24423978 PMC3963263

[2] Libé R Borget I Ronchi CL Zaggia B Kroiss M Kerkhofs T. Prognostic factors in stage III-IV adrenocortical carcinomas (ACC): an European Network for the Study of Adrenal Tumor (ENSAT) study. Ann Oncol Off J Eur Soc Med Oncol. (2015) 26:2119–25. doi: 10.1093/annonc/mdv329 26392430

[3] Cueto C Brown JH Richardson APJ. Biological studies on an adrenocorticolytic agent and the isolation of the active components. Endocrinology. (1958) 62:334–9. doi: 10.1210/endo-62-3-334 13512246

[4] Bergenstal DM Lipsett MB Moy RH Hertz ROY. Regression of Adrenal Cancer and Suppression of Adrenal Function in Man by o,p′-DDD11Read on behalf of Dr. D. M. Bergenstal, who died on September 12, 1959. In: Pincus G Vollmer EP, editors. Biological Activities of Steroids in Relation to Cancer. New York; London: Academic Press (1960). p. 463–75. doi: 10.1016/B978-1-4832-2866-2.50035-0

[5] Fassnacht M Assie G Baudin E Eisenhofer G de la Fouchardiere C Haak HR. Adrenocortical carcinomas and Malignant phaeochromocytomas: ESMO-EURACAN Clinical Practice Guidelines for diagnosis, treatment and follow-up. Ann Oncol Off J Eur Soc Med Oncol. (2020) 31:1476–90. doi: 10.1016/j.annonc.2020.08.2099 32861807

[6] Fassnacht M Dekkers O Else T Baudin E Berruti A de Krijger R. European Society of Endocrinology Clinical Practice Guidelines on the management of adrenocortical carcinoma in adults, in collaboration with the European Network for the Study of Adrenal Tumors. Eur J Endocrinol. (2018) 179:G1–G46. doi: 10.1530/EJE-18-0608 30299884

[7] Haider MS Ahmad T Groll J Scherf-Clavel O Kroiss M Luxenhofer R. The challenging pharmacokinetics of mitotane: an old drug in need of new packaging. Eur J Drug Metab Pharmacokinet. (2021) 46:575–93. doi: 10.1007/s13318-021-00700-5 PMC8397669 34287806

[8] Berruti A Baudin E Gelderblom H Haak HR Porpiglia F Fassnacht M. Adrenal cancer: ESMO Clinical Practice Guidelines for diagnosis, treatment and follow-up. Ann Oncol Off J Eur Soc Med Oncol. (2012) 23 Suppl 7:vii131–8. doi: 10.1093/annonc/mds231 22997446

[9] Puglisi S Calabrese A Basile V Pia A Reimondo G Perotti P. New perspectives for mitotane treatment of adrenocortical carcinoma. Best Pract Res Clin Endocrinol Metab. (2020) 34:101415. doi: 10.1016/j.beem.2020.101415 32179008

[10] van Koetsveld PM Creemers SG Dogan F Franssen GJH de Herder WW Feelders RA. The efficacy of mitotane in human primary adrenocortical carcinoma cultures. J Clin Endocrinol Metab. (2020) 105:407–17. doi: 10.1210/clinem/dgz001 PMC7006231 31586196

[11] Reidy-Lagunes DL Lung B Untch BR Raj N Hrabovsky A Kelly C. Complete responses to mitotane in metastatic adrenocortical carcinoma-A new look at an old drug. Oncologist. (2017) 22:1102–6. doi: 10.1634/theoncologist.2016-0459 PMC5599197 28559412

[12] Kroiss M Quinkler M Lutz WK Allolio B Fassnacht M. Drug interactions with mitotane by induction of CYP3A4 metabolism in the clinical management of adrenocortical carcinoma. Clin Endocrinol (Oxf). (2011) 75:585–91. doi: 10.1111/cen.2011.75.issue-5 21883349

[13] van Erp NP Guchelaar H-J Ploeger BA Romijn JA den Hartigh J Gelderblom H. Mitotane has a strong and a durable inducing effect on CYP3A4 activity. Eur J Endocrinol. (2011) 164:621–6. doi: 10.1530/EJE-10-0956 21220434

[14] Hermsen IG Fassnacht M Terzolo M Houterman S den Hartigh J Leboulleux S. Plasma concentrations of o,p’DDD, o,p’DDA, and o,p’DDE as predictors of tumor response to mitotane in adrenocortical carcinoma: results of a retrospective ENS@T multicenter study. J Clin Endocrinol Metab. (2011) 96:1844–51. doi: 10.1210/jc.2010-2676 21470991

[15] Puglisi S Calabrese A Basile V Ceccato F Scaroni C Altieri B. Mitotane concentrations influence outcome in patients with advanced adrenocortical carcinoma. Cancers (Basel). (2020) 12. doi: 10.3390/cancers12030740 PMC7140087 32245135

[16] Megerle F Herrmann W Schloetelburg W Ronchi CL Pulzer A Quinkler M. Mitotane monotherapy in patients with advanced adrenocortical carcinoma. J Clin Endocrinol Metab. (2018) 103:1686–95. doi: 10.1210/jc.2017-02591 29452402

[17] Altieri B Sbiera S Herterich S De Francia S Della Casa S Calabrese A. Effects of germline CYP2W1*6 and CYP2B6*6 single nucleotide polymorphisms on mitotane treatment in adrenocortical carcinoma: A multicenter ENSAT study. Cancers (Basel). (2020) 12. doi: 10.3390/cancers12020359 PMC7072643 32033200

[18] Weigand I Altieri B Lacombe AMF Basile V Kircher S Landwehr L-S. Expression of SOAT1 in adrenocortical carcinoma and response to mitotane monotherapy: an ENSAT multicenter study. J Clin Endocrinol Metab. (2020) 105:2642–53. doi: 10.1210/clinem/dgaa293 32449514

[19] Sbiera S Leich E Liebisch G Sbiera I Schirbel A Wiemer L. Mitotane inhibits sterol-O-acyl transferase 1 triggering lipid-mediated endoplasmic reticulum stress and apoptosis in adrenocortical carcinoma cells. Endocrinology. (2015) 156:3895–908. doi: 10.1210/en.2015-1367 26305886

[20] Holohan C Van Schaeybroeck S Longley DB Johnston PG. Cancer drug resistance: an evolving paradigm. Nat Rev Cancer. (2013) 13:714–26. doi: 10.1038/nrc3599 24060863

[21] Goyal Y Busch GT Pillai M Li J Boe RH Grody EI. Diverse clonal fates emerge upon drug treatment of homogeneous cancer cells. Nature. (2023) 620:651–9. doi: 10.1038/s41586-023-06342-8 PMC10628994 37468627

[22] Fassnacht M Terzolo M Allolio B Baudin E Haak H Berruti A. Combination chemotherapy in advanced adrenocortical carcinoma. N Engl J Med. (2012) 366:2189–97. doi: 10.1056/NEJMoa1200966 22551107

[23] Li H Durbin R. Fast and accurate short read alignment with Burrows-Wheeler transform. Bioinformatics. (2009) 25:1754–60. doi: 10.1093/bioinformatics/btp324 PMC2705234 19451168

[24] Picard toolkit. Broad Institute, GitHub Rrepos. (2019). Available at: https://broadinstitute.github.io/picard/.

[25] McKenna A Hanna M Banks E Sivachenko A Cibulskis K Kernytsky A. The Genome Analysis Toolkit: a MapReduce framework for analyzing next-generation DNA sequencing data. Genome Res. (2010) 20:1297–303. doi: 10.1101/gr.107524.110 PMC2928508 20644199

[26] Lai Z Markovets A Ahdesmaki M Chapman B Hofmann O McEwen R. VarDict: a novel and versatile variant caller for next-generation sequencing in cancer research. Nucleic Acids Res. (2016) 44:e108. doi: 10.1093/nar/gkw227 27060149 PMC4914105

[27] Cibulskis K Lawrence MS Carter SL Sivachenko A Jaffe D Sougnez C. Sensitive detection of somatic point mutations in impure and heterogeneous cancer samples. Nat Biotechnol. (2013) 31:213–9. doi: 10.1038/nbt.2514 PMC3833702 23396013

[28] Talevich E Shain AH Botton T Bastian BC. CNVkit: genome-wide copy number detection and visualization from targeted DNA sequencing. PloS Comput Biol. (2016) 12:e1004873. doi: 10.1371/journal.pcbi.1004873 27100738 PMC4839673

[29] Olshen AB Bengtsson H Neuvial P Spellman PT Olshen RA Seshan VE. Parent-specific copy number in paired tumor-normal studies using circular binary segmentation. Bioinformatics. (2011) 27:2038–46. doi: 10.1093/bioinformatics/btr329 PMC3137217 21666266

[30] Kim D Langmead B Salzberg SL. HISAT: a fast spliced aligner with low memory requirements. Nat Methods. (2015) 12:357–60. doi: 10.1038/nmeth.3317 PMC4655817 25751142

[31] Li B Dewey CN. RSEM: accurate transcript quantification from RNA-Seq data with or without a reference genome. BMC Bioinf. (2011) 12:323. doi: 10.1186/1471-2105-12-323 PMC3163565 21816040

[32] Ritchie ME Phipson B Wu D Hu Y Law CW Shi W. limma powers differential expression analyses for RNA-sequencing and microarray studies. Nucleic Acids Res. (2015) 43:e47. doi: 10.1093/nar/gkv007 25605792 PMC4402510

[33] Subramanian A Tamayo P Mootha VK Mukherjee S Ebert BL Gillette MA. Gene set enrichment analysis: a knowledge-based approach for interpreting genome-wide expression profiles. Proc Natl Acad Sci U.S.A. (2005) 102:15545–50. doi: 10.1073/pnas.0506580102 PMC1239896 16199517

[34] Ashburner M Ball CA Blake JA Botstein D Butler H Cherry JM. Gene ontology: tool for the unification of biology. The Gene Ontology Consortium. Nat Genet. (2000) 25:25–9. doi: 10.1038/75556 PMC3037419 10802651

[35] Aleksander SA Balhoff J Carbon S Cherry JM Drabkin HJ Ebert D. The gene ontology knowledgebase in 2023. Genetics. (2023) 224. doi: 10.1093/genetics/iyad031 PMC10158837 36866529

[36] Assié G Letouzé E Fassnacht M Jouinot A Luscap W Barreau O. Integrated genomic characterization of adrenocortical carcinoma. Nat Genet. (2014) 46:607–12. doi: 10.1038/ng.2953 24747642

[37] LaPensee CR Hammer GD. Targeting of a new node in lipid metabolism as a potential treatment strategy for ACC. Endocrinology. (2023) 164. doi: 10.1210/endocr/bqad003 36636840

[38] Warde KM Lim YJ Ribes Martinez E Beuschlein F O’Shea P Hantel C. Mitotane targets lipid droplets to induce lipolysis in adrenocortical carcinoma. Endocrinology. (2022) 163. doi: 10.1210/endocr/bqac102 PMC9342684 35797592

[39] Altieri B Ronchi CL Kroiss M Fassnacht M. Next-generation therapies for adrenocortical carcinoma. Best Pract Res Clin Endocrinol Metab. (2020) 34:101434. doi: 10.1016/j.beem.2020.101434 32622829

[40] Pozdeyev N Fishbein L Gay LM Sokol ES Hartmaier R Ross JS. Targeted genomic analysis of 364 adrenocortical carcinomas. Endocr Relat Cancer. (2021) 28:671–81. doi: 10.1530/ERC-21-0040 PMC8384129 34410225

[41] Crona J Beuschlein F. Adrenocortical carcinoma - towards genomics guided clinical care. Nat Rev Endocrinol. (2019) 15:548–60. doi: 10.1038/s41574-019-0221-7 31147626

[42] Kroiss M Sbiera S Kendl S Kurlbaum M Fassnacht M. Drug synergism of proteasome inhibitors and mitotane by complementary activation of ER stress in adrenocortical carcinoma cells. Horm Cancer. (2016) 7:345–55. doi: 10.1007/s12672-016-0273-2 PMC10355952 27631436

[43] Satoh K Zhang L Zhang Y Chelluri R Boufraqech M Nilubol N. Identification of niclosamide as a novel anticancer agent for adrenocortical carcinoma. Clin Cancer Res an Off J Am Assoc Cancer Res. (2016) 22:3458–66. doi: 10.1158/1078-0432.CCR-15-2256 PMC4947455 26873959

[44] Terzolo M Baudin AE Ardito A Kroiss M Leboulleux S Daffara F. Mitotane levels predict the outcome of patients with adrenocortical carcinoma treated adjuvantly following radical resection. Eur J Endocrinol. (2013) 169:263–70. doi: 10.1530/EJE-13-0242 23704714

[45] Lindhe O Skogseid B Brandt I. Cytochrome P450-catalyzed binding of 3-methylsulfonyl-DDE and o,p’-DDD in human adrenal zona fasciculata/reticularis. J Clin Endocrinol Metab. (2002) 87:1319–26. doi: 10.1210/jcem.87.3.8281 11889204

[46] Luo J Yang H Song B-L. Mechanisms and regulation of cholesterol homeostasis. Nat Rev Mol Cell Biol. (2020) 21:225–45. doi: 10.1038/s41580-019-0190-7 31848472

[47] Fedorova OV Zernetkina VI Shilova VY Grigorova YN Juhasz O Wei W. Synthesis of an endogenous steroidal na pump inhibitor marinobufagenin, implicated in human cardiovascular diseases, is initiated by CYP27A1 *via* bile acid pathway. Circ Cardiovasc Genet. (2015) 8:736–45. doi: 10.1161/CIRCGENETICS.115.001217 PMC4618091 26374826

[48] Cummins CL Volle DH Zhang Y McDonald JG Sion B Lefrançois-Martinez A-M. Liver X receptors regulate adrenal cholesterol balance. J Clin Invest. (2006) 116:1902–12. doi: 10.1172/JCI28400 PMC1483175 16823488

[49] Zheng S Cherniack AD Dewal N Moffitt RA Danilova L Murray BA. Comprehensive pan-genomic characterization of adrenocortical carcinoma. Cancer Cell. (2016) 29:723–36. doi: 10.1016/j.ccell.2016.04.002 PMC4864952 27165744

[50] Borges KS Pignatti E Leng S Kariyawasam D Ruiz-Babot G Ramalho FS. Wnt/β-catenin activation cooperates with loss of p53 to cause adrenocortical carcinoma in mice. Oncogene. (2020) 39:5282–91. doi: 10.1038/s41388-020-1358-5 PMC7378041 32561853

[51] Marullo R Werner E Degtyareva N Moore B Altavilla G Ramalingam SS. Cisplatin induces a mitochondrial-ROS response that contributes to cytotoxicity depending on mitochondrial redox status and bioenergetic functions. PloS One. (2013) 8:e81162. doi: 10.1371/journal.pone.0081162 24260552 PMC3834214

[52] Sandulache VC Chen Y Feng L William WN Skinner HD Myers JN. Metabolic interrogation as a tool to optimize chemotherapeutic regimens. Oncotarget. (2017) 8:18154–65. doi: 10.18632/oncotarget.15186 PMC5392315 28184025

[53] Muggia FM Bonetti A Hoeschele JD Rozencweig M Howell SB. Platinum antitumor complexes: 50 years since barnett rosenberg’s discovery. J Clin Oncol Off J Am Soc Clin Oncol. (2015) 33:4219–26. doi: 10.1200/JCO.2015.60.7481 26503202

[54] Reece DE Sullivan D Lonial S Mohrbacher AF Chatta G Shustik C. Pharmacokinetic and pharmacodynamic study of two doses of bortezomib in patients with relapsed multiple myeloma. Cancer Chemother Pharmacol. (2011) 67:57–67. doi: 10.1007/s00280-010-1283-3 20306195 PMC3951913

