# Supplementary material for: Pharmacogenomic analysis in adrenocortical carcinoma reveals genetic features associated with mitotane sensitivity and potential therapeutics
Source: Front Endocrinol (Lausanne). 2024 May 8;15:1365321. doi: 10.3389/fendo.2024.1365321 (PMC11109426; doi:10.3389/fendo.2024.1365321)
Supplement: Supplementary file 1 [file DataSheet_1.pdf]

Legends for Supplemental Figures and Tables

Supplementary Table 1. Clinical and tumor characteristics of patients receiving in vitro mitotane sensitivity testing

Supplementary Table 2. Compounds and highest concentrations used in HTS

Supplementary Figure 1. Correlation analysis between **(A)** *RRM1*, **(B)** *SOAT1*, **(C)** *CYP2W1* mRNA level and mitotane AUC.

Supplementary Table 1. Clinical and tumor characteristics of patients receiving in vitro mitotane sensitivity testing

Patient ID	Sex	Age	ENSAT stage	Functionality	Sample location	Ki67 index	SF-1 immuno-staining	Weiss score	Mitotane pretreatment
P1	F	52	III	Cortisol	Primary site	10%	+	7	No
P2	M	74	II	Non-functional	Primary site	10%	+	5	No
P3	F	22	IV	Non-functional	Metastatic, liver	NA	+	NA	Yes
P4	F	62	IV	Androgen	Metastatic, lung	50%	+	8	Yes
P5	F	54	IV	Non-functional	Metastatic, lung	15%	+	NA	No
P6	F	28	IV	Non-functional	Metastatic, lung	10%	+	NA	No
P7*	M	24	IV	Non-functional	Metastatic, abdominal cavity	NA	+	6	Yes
P8	F	70	IV	Cortisol	Recurrent, retroperitoneum	60%	+	NA	No
P9	F	66	IV	Androgen	Metastatic, liver	NA	+	NA	Yes
P10	F	63	IV	Non-functional	Metastatic, liver	20%	+	NA	Yes
P11	F	34	IV	Cortisol	Metastatic, liver	20%	+	NA	No
P12*	F	20	IV	Cortisol	Recurrent, retroperitoneum	10%	+	NA	No
P13	F	59	III	Cortisol, Androgen	Primary site	40%	+	7	No
P14	M	58	IV	Cortisol	Metastatic, paraspinal region	15%	+	7	Yes
P15	M	36	III	Aldosterone	Primary site	40%	+	8	No
P16	F	77	II	Non-functional	Primary site	40%	+	5	No
P17	M	76	II	Non-functional	Primary site	35%	+	5	No

* These patients received cytotoxic chemotherapy prior to surgical or biopsy intervention.

Abbreviations: ID, identity; F, female; M, male; NA, not available.

Supplementary Table 2. Compounds and highest concentrations used in HTS

Compound	Mechanism of action	Highest concentration in HTS (μM)	PMID
Sunitinib	Multi-targeted tyrosine kinase inhibitor	10	22654799
Sorafenib	Multi-kinase inhibitor	10	22673336
Cabozantinib	Multi-targeted tyrosine kinase inhibitor	10	31900481
Vandetanib	Multi-targeted tyrosine kinase inhibitor	10	32622829
Pazopanib	Multi-targeted tyrosine kinase inhibitor	10	32622829
Gefitinib	EGFR tyrosine kinase inhibitor	10	34410225
Lenvatinib	Multi-targeted tyrosine kinase inhibitor	10	32737143
Sulfatinib	Multi-targeted tyrosine kinase inhibitor	10	32622829; 28159938
Anlotinib	Multi-targeted tyrosine kinase inhibitor	50	32622829; 30231931
Apatinib	VEGFR-2 inhibitor	50	32622829; 26020064
Selumetinib	MEK1/2 inhibitor	10	30256438; 25385055
Dabrafenib	BRAF inhibitor	10	32622829; 24756796
Everolimus	mTOR inhibitor	1	28271381;21651476
Temsirolimus	mTOR inhibitor	1	28271381;21651476
Rapamycin	mTOR inhibitor	1	28271381;21651476
PI-103	PI3K and mTOR inhibitor	10	28271381;21651476
Gemcitabine	Deoxycytidine nucleoside analogue	10	24018612; 22257063
Temozolomide	DNA Alkylator	100	27603910
Capecitabine	Nucleoside antimetabolite/analog	100	20410174
5-Fluorouracil	Nucleoside antimetabolite/analog	50	18491247
Niclosamide	Antihelminthic agent	20	26873959
Dacarbazine	DNA Alkylator	20	25086465
Etoposide	Topoisomerase II inhibitor	10	22551107
Doxorubicin	Topoisomerase I inhibitor	10	22551107
Epirubicin	Topoisomerase inhibitor	1	9863019
Cisplatin	DNA Alkylator/Crosslinker	10	22551107

(+)-JQ-1	BET bromodomain inhibitor	1	36793283
Vorinostat	HDAC inhibitor	10	24238056
Tucidinostat	HDAC inhibitor	10	34600336
Tazemetostat	EZH2 inhibitor	10	27149985; 35369397
CPI-0610	BET inhibitor	10	36793283
Oxaliplatin	DNA Alkylator	10	15688617
Vismodegib	Hedgehog pathway inhibitor	10	34410225
2-Methoxyestradiol	Angiogenesis inhibitor	10	18491247
Retinoic acid	Agonist of RAR nuclear receptors	10	26885453
Bortezomib	Proteasome inhibitor	1	27631436
Palbociclib	CDK4 and CDK6 inhibitor	20	28265858
Disulfiram	Aldehyde-dehydrogenase (ALDH1) inhibitor	100	17026967
Olaparib	PARP inhibitor	100	34410225
Streptozocin	DNA alkylator	100	2949824

Supplementary Figure 1

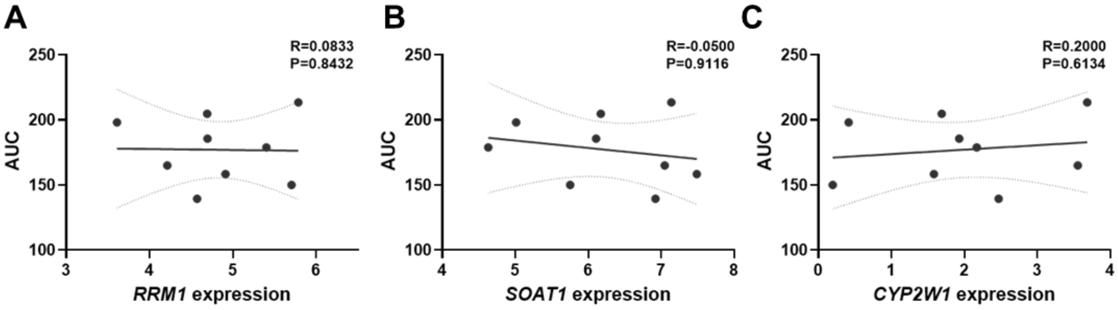

Supplementary Figure 1. Correlation analysis between **(A)** *RRM1*, **(B)** *SOAT1*, **(C)** *CYP2W1* mRNA level and mitotane AUC.
